# Overweight/obesity among school aged children in Bahir Dar City: cross sectional study

**DOI:** 10.1186/s13052-018-0452-6

**Published:** 2018-01-23

**Authors:** Teferi Mekonnen, Amare Tariku, Solomon Mekonnen Abebe

**Affiliations:** 1 Public Health Nutrition unit, School of Public Health, Bahir Dar University, P.O. Box 79, Bahir Dar, Ethiopia; 2 Departments of Human Nutrition, Institute of Public Health, University of Gondar, P.O. Box 196, Gondar, Ethiopia

**Keywords:** Overweight, Obesity, School aged children, Body mass index for age, Ethiopia

## Abstract

**Background:**

Developing countries, including Ethiopia are experiencing a double burden of malnutrition. There is limited information about prevalence of overweight/obesity among school aged children in Ethiopia particularly in Bahir Dar city. Hence this study aimed to assess the prevalence of overweight/obesity and associated factors among school children aged 6–12 years at Bahir Dar City, Northwest Ethiopia.

**Methods:**

A school based cross-sectional study was carried out. A total of 634 children were included in the study. Multi stage systematic random sampling technique was used.

A multivariable logistic regression analysis was used to identify factors associated with overweight/obesity. The association between dependent and independent variables were assessed using odds ratio with 95% confidence interval and *p*-value ≤0.05 was considered statistically significant.

**Results:**

The overall prevalence of overweight and/or obesity was 11.9% (95% CI, 9.3, 14.4) (out of which 8.8% were overweight and 3.1% were obese). Higher wealth status[adjusted OR = 3.14, 95% CI:1.17, 8.46], being a private school student [AOR = 2.21, 95% CI:1.09, 4.49], use of transportation to and from school [AOR = 2.53, 95% CI: 1.26,5.06], fast food intake [AOR = 3.88, 95% CI: 1.42,10.55], lack of moderate physical activity [AOR = 2.87, 95% CI: 1.21,6.82], low intake of fruit and vegetable [AOR = 6.45, 95% CI:3.19,13.06] were significant factors associated with overweight and obesity.

**Conclusion:**

This study revealed that prevalence of overweight/obesity among school aged children in Bahir Dar city is high. Thus, promoting healthy dietary habit, particularly improving fruit and vegetable intake is essential to reduce the burden of overweight and obesity. Furthermore, it is important to strengthen nutrition education about avoiding junk food consumption and encouraging regular physical activity.

## Background

Overweight/obesity is defined as abnormal or excessive fat accumulation that may impair health [1]. There is a substantial increase in prevalence of overweight/obesity among children and adolescents in both developed and developing countries [2]. Worldwide 43 million children were overweight/obese and, of which 35 million children are from developing countries. In addition, 92 million were found at risk of having overweight and obesity [3]. Studies done in developed countries revealed that the prevalence of overweight/obesity among school children is increasing [4–6].

In Africa, despite that there has been a higher burden of under nutrition, the magnitude of overweight and obesity is increasing at an alarming rate [2, 7–9]. In Sub-Saharan Africa, about 10.6% of school aged children were overweight/obesity, of which 2.5% were obese [10]. Pocket studies done in Tanzania and Kenya showed that the prevalence of overweight and obesity is also increasing in these two countries [11–13].

In Ethiopia, pocket studies done in Addis Ababa among school adolescent revealed that about 7.6–9.9% of school adolescent were overweight, while 0.9–2.8% were obese [14, 15].

Childhood obesity poses a major risk for serious diet-related chronic diseases, such as type 2 diabetes mellitus, cardiovascular disease, hypertension and stroke, and certain forms of cancer and, it is also noted to be a precursor of adverse health effects in adulthood, as overweight children are more likely to become overweight adolescents and adults [8, 16, 17]. Globally, an estimated 3.4 million deaths, 3.9% years of life lost, and 3.8% of Disability Adjusted Life Years (DALYs) are related to overweight and obesity [2]. The rising of overweight/obesity epidemics is attributed by rapid economic and epidemiologic transition, caused by several socioeconomic and demographic changes that reflects the profound changes in the society [7, 13, 18, 19].

In addition, overweight and obesity is favored by risky dietary behaviors such as consumption of fast food and drinks, eating away from home, skipping/missing of meal, regular drinking of sugar rich beverages and low serving/intake of fruit and vegetable [20–23]. In countries with limited resources and food availability, childhood overweight/obesity is favored by the good socioeconomic status of their parents while still insufficient nutrition education is available [24]. Eating behavior of the children is influenced by the availability of food, peers, siblings and parent’s behavior [25]. Sedentary behavior and physical inactivity are also important risk factors for childhood overweight and obesity [26].

Now days in Ethiopia the focus agenda is under nutrition, but there are evidences for nutrition transition particularly in urban cities which might have a contribution to over nutrition. In Ethiopia, particularly in the study area, there was no information regarding prevalence and contributing factors of overweight/obesity among school aged children, though it is known that early prevention of childhood obesity improves quality of life by decreasing an insult for chronic diseases for adolescent and adulthood life. Hence, it is important to assess the prevalence and associated factors of overweight/obesity among school aged children at the capital city of Amhara region, Bahir Dar city.

## Methods

### Study setting and population

This school based cross-sectional study was conducted from February 1st to April 15th, 2016 among first cycle elementary school students aged 6–12 years at Bahir Dar City. The city is the capital of Amhara National Regional State, which is situated in northwest Ethiopia. The city has 64 first cycle elementary schools of which 38 are public and 26 are private schools, with a total of 28,088 students. Of the total students, 6155 students were attending private schools while 21,933 were studying at public schools [27].

### Sample size and sampling procedures

Sample size was calculated using single proportion formula by considering the following assumptions: the prevalence of overweight/obesity among school aged children (6–12 years) was unknown and taken as 50%, margin of error of 5%, confidence level of 95%, 10% none-response rate, and a design effect of 1.5. The final sample size was 634.

A multi stage sampling technique was carried out. Primary sampling units were selected public and private schools. By taking 20% of both public and private school provides eight public and five private schools were included in the study. Lottery method was used to select schools, for each selected school, the samples were allocated proportional to number of students with respect to grade level. The secondary sampling unit was randomly selected students from each selected grade and section. Proportional allocation of the sample to the total number of students in each public and private were made and 139 students from private school and 495 students from public school with total of 634 students were included in the study using systematic random sampling technique.

### Data collection instrument and procedure

Structured pre-tested questionnaire adapted from the WHO stepwise questionnaire for Chronic Disease Risk Factor Surveillance were used for data collection [28]. The questionnaire was prepared in English first and then translated to local language, Amharic, and back to English by public health experts. Five clinical nurses as data collectors and two BSc nurses as supervisors were trained for data collection and supervision. Anthropometric measurements were done by trained data collectors using standard procedures and calibrated equipment.

The anthropometric assessment was done according to the standardized procedures stipulated by the Food and Nutrition Technical Assistance (FANTA) ‘Anthropometric Indicators Measurement Guide [29].

Height was measured to the nearest 0.1 cm in standing position at Frankfurt plane with the occipital, shoulder and the buttock touches the vertical stand using a stadiometer seca (Germany). Weight was measured to the nearest 0.1 kg using electronic weighing scale with wearing light clothes and with no shoes. The Percentile values for BMI-for-age (BAZ) of children were generated from WHO AnthroPlus version 1.0.3 software [30].

Overweight/obesity in school aged children were considered as dependant variable. BMI for age greater than or equal to 85th but less than 95th percentile was considered as overweight, while obesity was considered when BMI for age was greater than or equal to the 95th percentile [31].

### Data quality control

For data quality control a pre-test on 5% of the samples was performed and regular supervision during data collection was also carried out. The completeness of the questionnaire was checked before data entry too.

### Data processing and analysis

The data were first coded and entered using EpiData statistical software version 3.1 and then exported into SPSS statistical software version 20 for data management and analysis. Descriptive statistical analysis, such as simple frequencies and measures of central tendency was used to describe the characteristics of participants. Magnitude of overweight/obesity were determined by exporting age, sex, height, weight of the child to WHO AnthroPlus from SPSS software. Household wealth status was estimated using Household wealth index (HWI) constructed by using Principal Component Analysis (PCA) and the component score coefficient matrix was computed based on household assets then the households were categorized into tertiles according to the HWI as poor, middle income, high income.

Crud odds ratio with 95% CI was used to see the association between each independent variable and the outcome variable by using Binary logistic regression. Those associations with *p*-value < 0.2 were entered into the multivariable logistic regression model to control the effect of confounding. Variables with a p-value less than 0.05 were taken as statistically significant associated factors. The adjusted odds ratio with 95% confidence interval was presented to show the strength and precision of the association.

## Results

### Socio-demographic and economic characteristics

A total of 616 children participated, with a response rate of 97.2%. Of the total, 483 (78.4%) were attending public schools. 339 (55%) children were female. The mean age of the children was 9.7 ± 1.4 years. 519(84.3%) mothers were married and 475(88%) attended formal education. 321 (52.1%) and 224(36.4%) of fathers and mothers were self employed, respectively (Table 1).

**Table 1:** Socio-demographic characterstics of parents and children aged 6–12 years in Bahir Dar City northwest Ethiopia, (*n* = 616)

Variables	Frequency	Percentage
Marital status respondent		
Single	8	1.3
Married/partner	519	84.3
Divorced/separated	54	8.8
Widowed	35	5.7
Maternal religion		
Orthodox	500	81.2
Muslim	86	14.0
Catholic	8	1.3
Protestant	20	3.2
Others	2	0.3
Educational status of the mother		
No Formal Education	141	22.9
Primary Education	158	25.6
Secondary education &Above	317	51.5
Educational status of the father		
No Formal Education	124	20.1
Primary Education	127	20.6
Secondary education &Above	365	59.3
Fathers occupation		
private employed	321	52.1
Formally Employed	237	38.5
Daily laborer	58	9.4
Mothers occupation		
Private Employed	224	36.4
Formally Employed	131	21.3
Daily laborer	41	6.7
Housewife	220	35.7
Respondent sex		
Male	70	11.4
Female	546	88.6
Child sex		
Male	277	45
Female	339	55
Type of school		
Public	483	78.4
Private	133	21.6

### Type of school the students enrolled by the household’s wealth status

**Fig. 1:**
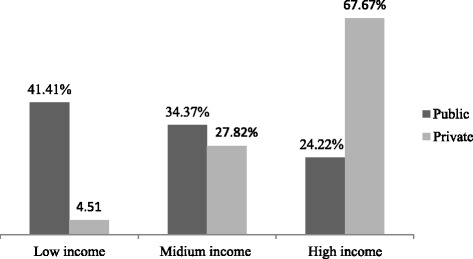
Type of school enrolment stratified by household wealth status among school children in Bahir Dar city, 2016

A substantial variation in the type of school enrollment was observed in the lowest and highest household’s wealth status category. Accordingly, about 41.4 and 4.51% of children from the lowest wealth status attended public and private schools, respectively. On the other hand, nearly two-third (67.67%) and one-quarter (24.22%) of children of the highest wealth status households were enrolled at private and public schools, respectively (Fig 1).

### Dietary habit

Regarding the dietary pattern of the children, 347 (56.3%) school aged children consumed both fruit and vegetable, 324 (52.6%) had snacks, 51.2% two times per week or less. With regard to meal frequency, 593 (96.3%) took meal for three times or more, and about 76.8% had bought and used either one or more of this food list(biscuit, chocolate, ice-cream or cake) listed under fast food intake. 285(46.3%) watches television while eating. Furthermore, 268(43.5%) had a habit of missing meals and 341(56.7%) had access to the restaurant/shop for fast food (Table 2).

**Table 2:** Dietary habit of school children aged 6–12 years in Bahir Dar city northwest Ethiopia, (*n* = 616)

Characteristics’		
Fruit and vegetable consumption	Frequency	Percentage (%)
Yes	347	56.3
No	269	43.7
Uses snack		
Yes	324	52.6
No	292	47.4
Number of snacks/day		
≤ 2 snacks/day	166	51.2
≥ 3snacks/day	158	48.8
Number of meals/day		
< 3 meals/day	23	3.7
≥ 3 meals/day	593	96.
Ways of getting lunch		
Taking from home	600	97.6
cafeteria/school Lounge	16	2.4
Intake of fast food		
Yes	473	76.8
No	143	23.2
Watching Television while eating		
Yes	285	46.3
No	331	53.7
Eating while reading		
Yes	107	17.4
No	509	82.6
Missing of meals		
Yes	268	43.5
No	348	56.6
Access to restaurant/shop for fast food		
Yes	349	56.7
No	267	43.3

### Physical activity and sedentary behavior related characteristics

Regarding physical activity and sedentary behavior, the majority of the children (82.1%) used to walk to school and from school. 110 (17.9%) used public transportation to and from school. Nearly one fourth (25.5%) of the children participated in sport activities, out of which 141 (88.7%) performs for more than 30 min. 274 (44.5%) participated in doing an extra home activities. Nearly one-fifth of school aged children (20.6%) performed moderate physical activities.

Fifty eight (9.4%) children used to play computer or mobile games. 527(85.6%) used to watch television at home, around two-third (61.4%) had watched TV more than 1 h per day (Tables 3 and 4).

**Table 3:** Physical activity related behavior of school children aged 6–12 years in Bahir Dar city northwest Ethiopia 2016, (*n* = 616)

Characteristics	Number	Percentage (%)
Form of transport used for going to school		
Walking	506	82.1
Transportation	110	17.9
Sport activity		
Yes	157	25.5
No	459	74.5
Number minutes at sport activity		
< 30 min	17	11.3
≥ 30 min	140	88.7
Extra home activity		
Yes	274	44.5
No	342	55.5
Doing an extra home activity		
< 30 min	52	19.0
≥ 30 min	222	81.0
Computer game\mobile game		
Yes	58	9.4
No	558	90.6
Time at computer\mobile game		
≤ 60 min	45	77.6
> 60 min	13	22.4
Watching Television		
Yes	527	85.6
No	89	14.4
Time at television/day		
< 60 min	149	28.3
≥ 60 min	378	71.7
Performing moderate activity		
Yes	127	20.6
No	489	79.4

**Table 4:** Prevalence of overweight and/obesity among school children in Bahir Dar city northwest Ethiopia, 2016 (*n* = 616)

	Classification	Prevalence (%)	Confidence interval
Body mass index for age	Overweight	8.8	6.5–11
	Obesity	3.1	1.8–4.5
	Overweight/obesity	11.9	9.3–14.4

### Anthropometric measurements and prevalence of overweight and obesity

The mean weight (±SD) and height (±SD) of the surveyed children was 28 (±6) kilograms and 129.4 (±8.8) centimeters, respectively.

The overall prevalence of overweight/obesity using BMI for age cut off was found to be 11.9% (95% CI: 9.3, 14.4), out of which about 8.8 and 3.1% of children were overweight and obese, respectively. The proportion of overweight/obesity among private school children was 29.3%, while in public schools it was 7%. The sex specific proportion of overweight/obesity among male private and public school students was 26.9 and 5.6%, respectively, and the proportion of overweight/obesity among female private and public school students was 31.43 and 8.18%, respectively.

### Factors associated with overweight/obesity among school aged children

The multivariable logistic regression analysis revealed that fruit and vegetable intake, household wealth status, type of school, physical activity, mode of transportation, fast food intake were significantly associated with overweight/obesity.

Accordingly, the odds of being overweight/obese were 6.5 times higher in children who had low fruits and vegetables consumption [AOR = 6.45, 95% CI: 3.19, 13.06].

Similarly, the higher likelihood of being overweight/obese was found among children who belonged to the higher household wealth tertile compared to children from the lower tertile [AOR = 3.14, 95% CI: 1.17, 8.46].

The higher odds of overweight/obesity were noted among children attending private schools compared to public school students [AOR = 2.21, 95% CI: 1.09, 4.49]. Likewise, increased odds of overweight/obesity was observed among children who used transportation to and from school [AOR = 2.53, 95% CI: 1.26, 5.06] as well as those who did not perform moderate physical activity [AOR=, 2.87, 95% CI 1.21, 6.82]. The odds of being overweight/obesity were higher among children who had taken fast food compared to those children who didn’t take fast food [AOR = 3.88, 95% CI 1.42, 10.55] (Table 5).

**Table 5:** Factors associated with overweight/obesity among school children in Bahir Dar City North West Ethiopia, 2016, multivariable analysis (*n* = 616)

Variables	Overweight or obesity		COR (95% CI)	AOR (95% CI)
	Yes	No		
Maternal level of education				
Secondary and above	54	263	3.93 (1.74,8.87)^*^	1.42 (0.53,3.83)
Primary	12	146	1.57 (0.60, 4.11)	1.24 (0.42,3.69)
No formal education	7	134	1.0	
Husband/partner occupation				
Private employed	31	290	1.44 (0.49,4.25)	1.05 (0.56,1.98)
Formally employed	38	199	2.58 (0.88,7.54)^*^	1.90 (0.52,6.89)
Daily laborer	4	54	1.00	
Fruit and vegetable intake				
Yes	11	336	1.00	
No	62	207	9.15 (4.71,17.78)^**^	6.45 (3.19,13.06)^**^
Mode of transport				
Walking/Bicycle	38	468	1.00	
Transportation	35	75	5.75 (3.42,9.67)^**^	2.53 (1.26,5.06)^*^
Fast food intake				
Yes	68	405	4.63 (1.83, 11.73)^*^	3.88 (1.42,10.55)^*^
No	5	138	1.00	
Household wealth status				
Low income	7	199	1.00	
Middle income	17	186	2.60 (1.05,6.41)^*^	1.83 (0.66,5.06)
High income	49	158	8.82 (3.89,20.00)^**^	3.14 (1.17,8.46)^*^
Watching television				
Do not watch TV	3	86	1.00	
Watching For < 1 h	5	144	1.00 (0.23,4.27)	0.45 (0.10,2.11)
Watching For ≥1 h	65	313	5.95 (1.83,19.41)^*^	1.65 (0.45,6.10)
Type of school				
Public	34	449	1.00	
Private	39	94	5.48 (3.29,9.13)^**^	2.21 (1.09,4.49)^*^
Missing meal				
Yes	38	230	1.48 (0.91, 2.41)	1.11 (0.61,2.01)
No	35	313	1.00	
Performing Moderate physical activity				
Yes	10	117	1.00	
No	63	426	1.73 (0.86,3.48)	2.87 (1.21,6.82)^*^
Age of the child				
Age < 10 years	39	250	0.74 (0.46,1.21)	1.22 (0.66,2.56)
Age ≥ 10 years	34	293	1.00	

*CI* confidence interval, *COR* crude odds ratio, *AOR* adjusted odds ratio

^*^p-value < 0.05; ^**^p-value< 0.001

## Discussion

This study revealed that 11.9% of school aged children are overweight/obesity with [95% CI: 9.3, 14.4)]. This prevalence was consistent with the study done in Addis Ababa, Ethiopia (12.7%) [15], and the study done in Sub-Saharan Africa countries (10.6%) [10]. The finding of this study was also in the range of the study reports from Kenya in which 14.4% school children were overweight [32] and different parts of India, according to which about 4.9–12% of children were overweight and obesity [33–35].

This could be related to contextual similarities in change in dietary habit and lifestyle related to increased urbanization and nutrition transition. In addition, though there was a difference in age of the study participants between the current and the previous study settings, the similarity in the prevalence could be due to the fact that childhood overweight/obesity is one of the risk factors for adolescent overweight/obesity in low and middle income countries [16].

However, the prevalence of overweight/obesity was lower compared to the reports from other African countries, such as Dares Salaam, Tanzania (15%) [11], Nairobi, Kenya 19% [13],and Egypt (31.4%) [9]. The discrepancy might be due to the disparities in study population and dietary habit.

Children who were from high income families were more likely to be overweight/obese as compared to low income families’ children. This might be due to the fact that an increase in wealth status might shift high income families into nutrition transition by replacing traditional diet with energy dense diet and sedentary life style which are known risk factors of overweight/obesity [16].

Private school children’s’ were more likely to be overweight/obese as compared public school. This finding was supported by the findings of other studies that assessed children and adolescent’s overweight/obesity in Tanzania, Kenya, Puerto Rico and India [8, 12, 13, 19, 36]. This might be due to the fact that in Ethiopia the public schools are funded by the government, where as private schools charge considerably higher tuition fees. Given that, only parents who can afford the fees would choose to enroll their children in private schools. We know that in developing countries children from high income families are more likely to have nutrition transition and sedentary behavior.

The odds of being overweight/obesity were higher among children who had taken fast food compared to those children who didn’t take any fast food during the week. Similar findings were reported in Addis Ababa, London, and America [15, 20–22]; this might be related to the higher energy content of most of the fast foods.

Our study also indicated that low fruit and vegetable intake was associated with higher risk of being overweight/obese. The finding was in agreement with the previous studies from Equatorial Guinea, London, Saudi Arabia [20, 22, 23]. This could be due to the fact that their bulk and low energy density of fruit and vegetable (with high amount of water and fiber) are believed to reduce energy dense food consumption and helps to easily attain satiety.

Children who used transportation to and from school were more likely to be overweight/obese than those who used to walk or to cycle to and from school. Similar findings are reported in Addis Ababa and Nigeria [15, 37]. It is known fact that the use of transportation leads to sedentary behaviour by decreasing energy expenditure. Children who had no moderate physical activities were more likely to become overweight/obese as compared to children who had performed moderate physical activities, which is supported by the previous studies [22, 37]. Physical activity determines number of calories that are spent or stored in the body as fat and maintains healthy weight status because of its potentially major impact on body composition, metabolism, and increasing energy expenditure.

### Study limitation

Firstly, the study is not free from the pitfalls of cross sectional study design. Consequently, the result of the study didn’t show the temporal cause and effect relationship between the outcome and the independent variables. Recall bias may be the second limitation of the study mainly in measuring the child’s dietary habit and level of physical activity.

## Conclusion

This study provided that Overweight/obesity among school aged children in Bahir Dar City is high. The result of an adjusted analysis showed that fruit and vegetable intake, household wealth status, type of school. Physical activity and mode of transportation were identified as the key determinants of overweight and obesity. Therefore, promoting a healthy life style such as improving fruit and vegetable intake and regular physical activity are essential. In addition, special attention needs to be given for children from high-income families and enrolled in private schools.

## Abbreviations

AOR: Adjusted odds ratio
BMI: Body mass index
CDC: Center for diseases control and prevention
COR: Crude odds ratio
DALYs: Disability adjusted life years
DHS: Demographic Health survey
EDHS: Ethiopian Demographic Health Surveys
SD: Standard deviation
WHO: World Health Organization
